# The Efficacy of *Lactobacillus delbrueckii* ssp. *bulgaricus* Supplementation in Managing Body Weight and Blood Lipids of People with Overweight: A Randomized Pilot Trial

**DOI:** 10.3390/metabo14020129

**Published:** 2024-02-16

**Authors:** Pei-Yi Chu, Ying-Chun Yu, Yi-Cheng Pan, Yun-Hao Dai, Juan-Cheng Yang, Kuo-Chin Huang, Yang-Chang Wu

**Affiliations:** 1 Chinese Medicine Research and Development Center, China Medical University Hospital, Taichung 404327, Taiwan; ositachucmu@gmail.com (P.-Y.C.); alce2943@gmail.com (Y.-C.P.); s8355099@gmail.com (Y.-H.D.); qq9113054@gmail.com (J.-C.Y.); 2 Department of Medical Research, Chinese Medicine Research and Development Center, China Medical University Hospital, Taichung 404327, Taiwan; yingchun.ycyu@gmail.com; 3 Graduate Institute of Biomedical Sciences, Center for Tumor Biology, School of Medicine, China Medical University, Taichung 404328, Taiwan; 4 Ph.D. Program for Cancer Biology and Drug Discovery, China Medical University and Academia Sinica, Taichung 404328, Taiwan; 5 School of Pharmacy, China Medical University, Taichung 404328, Taiwan; 6 School of Chinese Medicine, College of Chinese Medicine, China Medical University, Taichung 404328, Taiwan; 7 Department of Chinese Medicine, China Medical University Hospital, Taichung 404327, Taiwan; 8 Graduate Institute of Integrated Medicine, College of Chinese Medicine, China Medical University, Taichung 404328, Taiwan; 9 Department of Medical Laboratory Science and Biotechnology, College of Medical and Health Science, Asia University, Taichung 413305, Taiwan

**Keywords:** *Lactobacillus delbrueckii* ssp. *bulgaricus*, body weight, triglyceride, lipoprotein, obesity

## Abstract

This study aimed to evaluate the efficacy of *Lactobacillus delbrueckii* ssp. *bulgaricus* (*L. bulgaricus*) in improving body weight, obesity-related outcomes, and lipid profiles of overweight people. Thirty-six overweight participants were randomly assigned to either a probiotic or a placebo group. A placebo powder or *L. bulgaricus* powder (containing 1 × 10^8^ colony-forming unit (CFU) of the probiotic) was administered daily for 12 weeks. Body composition was determined, and blood tests were performed before and after the intervention. *L. bulgaricus* supplementation under the present condition did not affect the body weight, fat percentage, or body mass index (BMI) of the participants, while it resulted in a notable decrease in blood triglyceride (TG) levels, which corresponded to a lowering of the TG proportion in the composition of large VLDL (L–XXL sized fractions) and HDL (M and L fractions) in the probiotic-treated group. These results suggest that *L. bulgaricus* supplementation under the current conditions may not be helpful for losing weight, but it has the potential to decrease blood TG levels by modulating TG accumulation in or transport by VLDL/HDL in obese patients. *L. bulgaricus* supplements may have health-promoting properties in preventing TG-related diseases in overweight people.

## 1. Introduction

In recent years, probiotic supplements have become increasingly popular in daily life for preventing diarrhea, easing vaginal and urinary infections, or preventing autoimmune diseases [1,2,3]. Probiotics cause the formation of a properly balanced gut bacterial population, with a balance between pathogens and the bacteria necessary for the normal functioning of the organism, and may improve immune system function and nutrient absorption. Recently, the gut microbiota was found to be involved in various metabolic pathways and energy balance regulation [4,5]. The composition of the gut microbiota differs between obese- and normal-weight individuals [6,7]. Dietary alteration of the gut microbiome is a target for treating obesity [8]. However, clinical trials examining the influence of probiotics on obesity-related factors have yielded inconsistent outcomes. Some studies have demonstrated weight loss in participants supplemented with *lactobacilli* and *bifidobacteria*, even while maintaining their normal diet and lifestyle over the study’s duration [9,10]. In a 12-week trial, participants consuming *L. gasseri* experienced significant reductions in visceral fat, body mass index, waist and hip circumference, and body fat mass compared to the control group [11]. Conversely, another trial involving *L. rhamnosus* supplementation alongside an energy-restricted diet did not significantly impact weight loss in all participants but showed a reduction in weight in females [12]. A systematic review encompassing 19 randomized trials with 1412 participants revealed diverse outcomes [13]. Some studies report significant decreases in body weight and/or body fat with probiotics, while others indicate no effect or even increased body weight. It suggests that specific probiotics have the potential to serve as health supplements for treating or preventing obesity and overweight.

*Lactobacillus* is a type of lactic acid bacterium commonly found in the human gastrointestinal tract and in fermented dairy products such as cheese, yogurt, and kefir [14]. The isolation of specific probiotic strains for targeted application is a strategy for improving the efficacy of probiotic supplements. Some species of *Lactobacillus*, such as *L. gasseri* and *L. rhamnosus*, have been evaluated for inducing weight loss [15]. *Lactobacillus delbrueckii* ssp. *bulgaricus* (*L. bulgaricus*) is one of the most common lactobacilli starters used in the manufacture of a large variety of fermented milk products. *L. bulgaricus* is reported to exhibit an outstanding inhibitory effect on pancreatic lipase activity in vitro and effectively managed the fat and weight accumulation and reversed the increased blood lipid, sugar, and insulin levels caused by a high-fat diet in mice [16]. Our hypothesis posits that *L. bulgaricus* can contribute to the reduction in body weight and alleviate health risk factors associated with obesity in overweight individuals. Thus, the objective of this study was to examine the effectiveness of daily *L. bulgaricus* supplementation in weight management among overweight participants. The primary goal is to evaluate its impact on lowering body weight and addressing health risk factors related to obesity, including blood lipid levels, sugar regulation, and insulin levels.

## 2. Methods and Design

### 2.1. Study Approval

This study was approved by China Medical University Hospital Research Ethics Committee on 31 May 2021 (NO. CMUH110-REC2-070) and registered with www.clinicaltrials.gov, accessed on 31 May 2021 (NCT06244186). It was carried out at Chinese Medical University Hospital in Taiwan. This was a single-center, randomized, double-blind, placebo-controlled trial and was conducted in accordance with the principles of the Declaration of Helsinki. 

### 2.2. Participants

For the pilot trial, 36 participants were recruited. The participants were recruited from the patients of the Endocrinology and Metabolism Department at China Medical University Hospital, followed by the verification of inclusion and exclusion criteria through interviews. The inclusion criteria comprised: (1) age ≥ 20 years old; (2) overweight (BMI ≥ 23) or body fat percentage ≥ 25% for males and ≥30% for females; (3) having any risk factors such as atherosclerotic cardiovascular disease (ASCVD), type 2 diabetes, age ≥ 45 years for males, ≥55 years for females or postmenopausal, hypertension, dyslipidemia (total cholesterol (CHOL) > 200 mg/dL or low-density lipoprotein cholesterol (LDL-C) > 130 mg/dL or triglycerides (TG) > 130 mg/dL), and high-density lipoprotein cholesterol (HDL-C) < 40 mg/dL; (4) if routinely taking medication for lowering blood glucose, blood pressure, or lipid levels, there should not be significant dosage changes within the past three months; (5) and being willing to participate after receiving an explanation from the physician, completing the trial plan, and signing the consent form. The exclusion criteria included the following: (1) history of diabetic ketoacidosis; (2) medical records indicating the occurrence of cerebrovascular disease, acute myocardial infarction, coronary artery bypass surgery, placement of coronary artery stents, or peripheral vascular disease within the last 6 months; (3) occurrence of acute infectious diseases within the last month and antibiotic use for >7 days; (4) short-term use of steroids, non-steroidal anti-inflammatory drugs (NSAIDs), immunosuppressive drugs, interferons, immunomodulators, or any changes in the dose of long-term medications within the last month; (5) use of any weight-loss drugs in the last three months (including orlistat, lorcaserin, and liraglutide); (6) history of any cancer or undergoing cancer treatment in the past 5 years; (7) abnormal liver function (glutamic-oxaloacetic transaminase (GOT) or glutamic-pyruvic transaminase (GPT) greater than 3 times the normal upper limit) or liver cirrhosis; (8) impaired kidney function (eGFR < 30 mL/min/1.73 m^2^); (9) history of alcohol abuse; (10) participation in any other interventional clinical research within the last month; (11) pregnant and breastfeeding women; (12) history of allergy to the investigational product; (13) and participants deemed unsuitable for inclusion by the principal investigator.

### 2.3. Study Design and Intervention

The study started in September 2021, and follow-up was completed in November 2022. The enrolled participants were randomly divided into the probiotics group or placebo group. To randomly allocate the participants into one of the two groups, simple randomization was performed through manual drawing of lots by a chief researcher. The participants and research assistants were blinded to allocation. Seventeen and nineteen participants were, respectively, assigned to the placebo group and the probiotics group. The probiotics we used were in the form of 100 mg L. bulgaricus powder packets, each containing 1 × 10^8^ CFU of probiotics (TCI CO., Taipei, Taiwan). Initially (day 1), the participants underwent measurements for weight, body fat, and BMI, and fasting blood samples were collected. Subsequently, each participant received one package of either a probiotic or a placebo sample, along with drinking water on the same day. From that point onward, the participants self-administered one package of the sample daily for 12 weeks, and the participants’ sample consumption and physical conditions were tracked by the research assistants weekly. If a doctor determines adverse reactions or unsuitability for the trial, the trial of the participant will be discontinued, and the data will be excluded.

Throughout the study duration, the participants maintained their regular diet and lifestyle. At the endpoint (day 84), weight, body fat, and BMI were measured, and fasting blood samples were collected again. Weight and body fat were measured with Karada Scan 216 (OMRON, Kyoto, Japan). Alanine aminotransferase (ALT), aspartate aminotransferase (AST), blood urea nitrogen (BUN), creatinine (CRE), CHOL, HDL-C, LDL-C, TG, hemoglobin A1c (HbA1c), and glucose-ante cibum (Glu-AC) levels were analyzed and reported by Laboratory Medicine at Chinese Medical University Hospital (Taichung, Taiwan). Data were expressed as the mean ± standard error of the mean (SEM). Statistical significance between the groups was carried out by student-paired *t*-tests or Mann–Whitney test. *p*-values lower than 0.05 were considered statistically significant.

### 2.4. Metabolite Quantification and Metabolome Data Analysis

Nightingale’s biomarker analysis/platform was also executed on fasting blood samples taken from the patients before and after the placebo/probiotic intervention (Nightingale Health Ltd., Helsinki, Finland; nightingalehealth.com/). The details of the studies have already been disclosed [17]; this platform is used for quantifying and identifying metabolites based on NMR spectra. Lipoprotein subclass profiling with lipid concentrations and composition, different cholesterol and triglyceride measurements, various fatty acids, etc., are detected and declared as mmol/mol, percentage (pct or), or ratio. Further differential expressed analysis was conducted on the significant differences between the intergroup (probiotics versus placebo) and intragroup (before and after the intervention). Representative charts and plots (lollipop chart, dot plot, and hierarchical clustering) can be implemented based on the results of differentially expressed analysis. The statistical significance was calculated using two-way ANOVA as post hoc tests (*, *p* ≤ 0.05; **, *p* ≤ 0.01; ***, *p* ≤ 0.001). If no asterisk is shown, it represents no significance between the comparison.

## 3. Results

### 3.1. Subject Disposition and Characteristics

**Figure 1:**
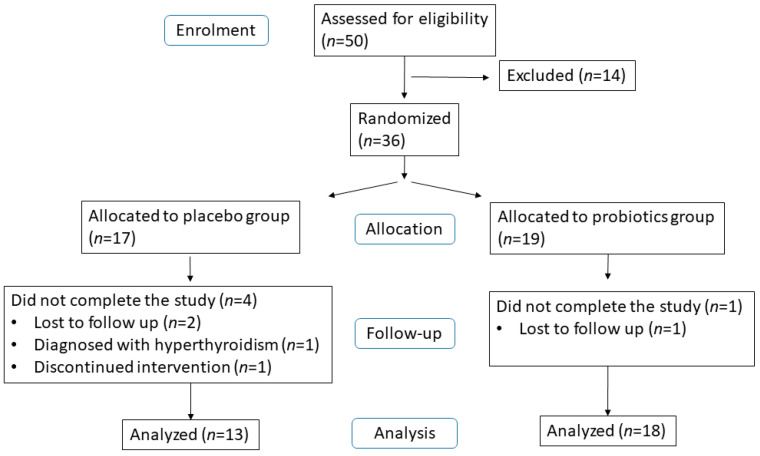
Flow diagram of the study.

Fifty overweight patients were assessed in this study. Thirty-six patients meeting the inclusion criteria were randomly allocated to the placebo group or the probiotics group. Four patients from the placebo group were excluded from the study due to being lost to follow-up, being diagnosed with hyperthyroidism, or discontinuing the intervention. Two patients from the probiotics group were excluded due to being lost to follow-up. At the end of the intervention, 31 patients completed the study (Figure 1). The baseline characteristics of the two groups were similar (Table 1).

### 3.2. Efficacy Analysis

Body weight, body fat, and BMI are the main parameters used to evaluate the efficacy of weight management. In this study, the changes in body weight, fat, or BMI did not differ significantly between the two groups (Table 2). Twelve weeks of supplementation with *L. bulgaricus* failed to induce weight loss in the subjects.

In addition, no significant differences in changes in blood biomedical parameters, ALT, BUN, CRE, and C-reactive protein (CRP) levels, were observed between the two groups (Table 3). *L. bulgaricus* probiotics led to a decrease in AST levels compared to the placebo group (*p* = 0.0147). However, the difference between preintervention and postintervention AST levels of the probiotics group was not significant (*p* = 0.1196). No significant difference in the change in Glu-AC, HbA1c, or insulin levels was observed between the probiotics and placebo groups. Blood lipid levels were also measured. No significant differences in the changes in CHOL or HDL levels were observed between the two groups. The LDL-C level decreased about 12.04 ± 6.323 units after intervention in the placebo group (*p* = 0.0417). In contrast, its level did not decrease in the probiotics group (*p* = 0.0937). TG levels decreased after intervention in the probiotics group (−21.00 ± 11.61, *p* = 0.0447), and the difference in the change in the TG level between the two groups was significant (*p* = 0.0412). The Alb level increased after intervention in the probiotics group (*p* = 0.0353). However, the difference in the change in the Alb level between the probiotics and placebo groups was not statistically significant (*p* = 0.3287).

### 3.3. Findings of Metabolome Lipid Profiling

Seven participants in the placebo group and nine participants in the probiotics group were randomly selected for Nightingale metabolome analysis. These individuals provided fasting serum samples both before and after the intervention. Differential expression (DE) analysis was performed to identify significant metabolites associated with the *L. bulgaricus* intervention. The magnitude of the change, which is related to the numeric values of the log2-fold changes, included both upregulation (indicated by positive values) and downregulation (indicated by negative values). The magnitude of the log2-fold change significantly distinguishes the change from those observed in other comparisons. According to the baseline comparison result (Figure S1), participants in the probiotics group may exhibit a more severe metabolic profile compared to those in the placebo group in the beginning. Therefore, we focused on the changes within each group before and after the intervention.

### 3.4. Differential Expression Analysis

**Figure 2:**
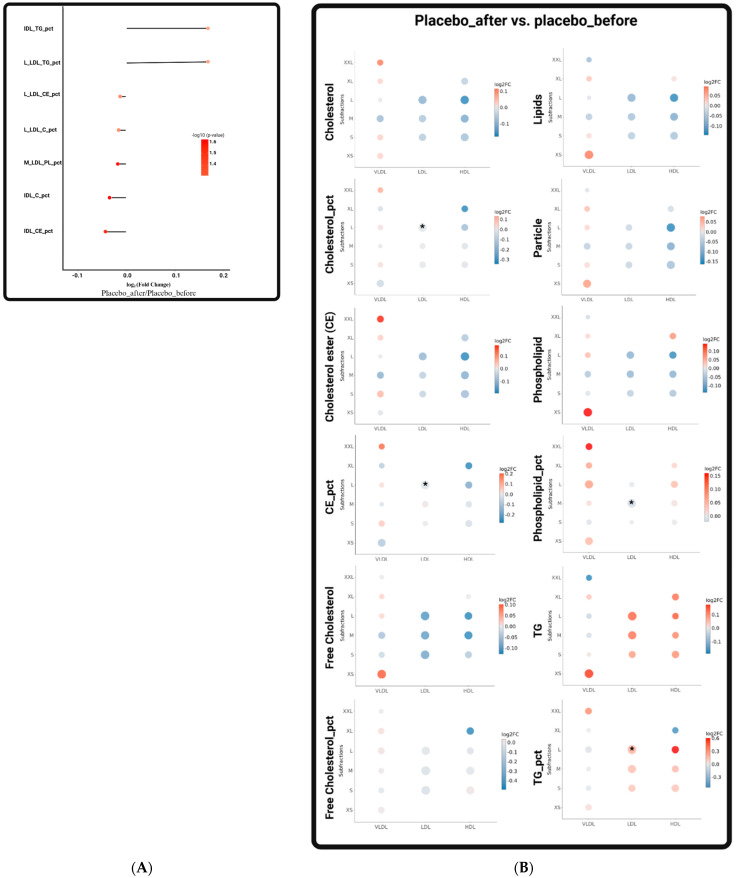
Significant changes in metabolites in the placebo group after intervention. (**A**) The lollipop chart shows the log2-fold change in lipid metabolites in participants before and after intervention in the placebo group (placebo after vs. placebo before). (**B**) Comparison of changes in lipoprotein subclasses before and after placebo intervention. Dot size represents the magnitude of the *p*-value of significance, and dot color indicates the log2-fold change in expression within lipoprotein classes. Abbreviations: Size_Liproprotein_Lipid_pct, the specific lipid-to-total lipids ratio in lipoprotein size subfractions (Size: XXL, extremely large; XL, extra-large; L, large; M, medium; S, small. Lipoprotein: IDL, intermediate-density lipoprotein; LDL, low-density lipoprotein; HDL, high-density lipoprotein. Lipid: TG, triglyceride; C, cholesterol; CE, cholesteryl ester; PL, phospholipid). Statistically significant at *, *p* ≤ 0.05.

In the placebo group, seven metabolic parameters showed significant changes after the 12-week placebo intervention. These changes are the alterations in lipid proportion within various lipoproteins, including an increase in the TG proportion in intermediate-density lipoprotein (IDL) and large LDL (L_LDL), a decrease in the cholesteryl ester (CE) and cholesterol (C) proportions in L_LDL and IDL, and a decrease in the phospholipid (PL) proportion in medium LDL (M_LDL) (Figure 2). The placebo intervention seemed to improve several lipid indicators in the participants. These findings may be attributed to factors unrelated to the placebo effect or other confounding variables in the study.

**Figure 3:**
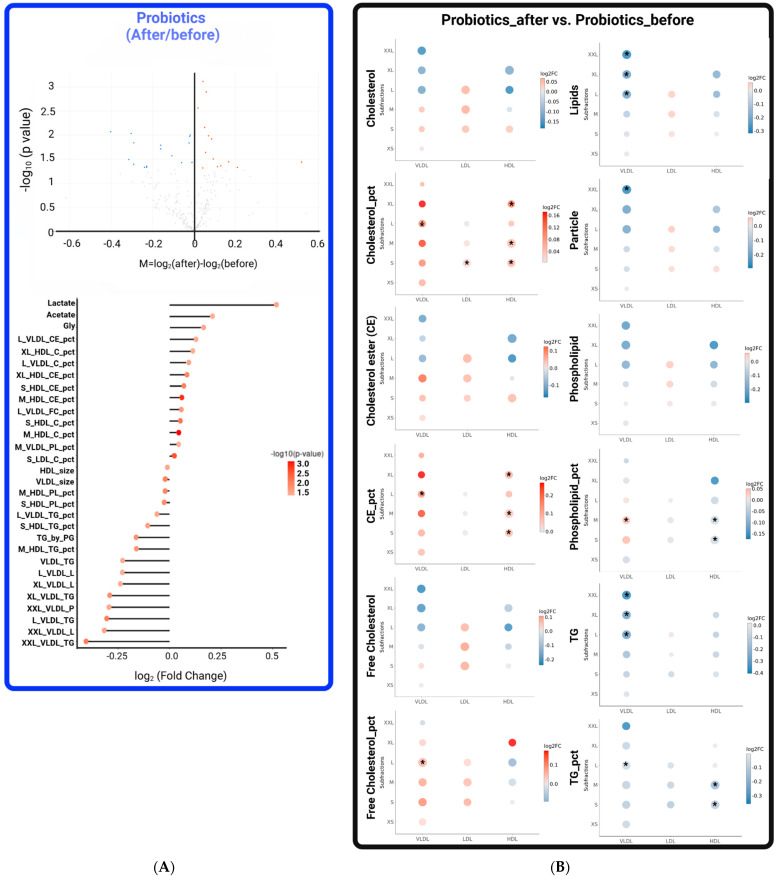
Significant changes in metabolites in the probiotics group after intervention. (**A**) The upper panel displays a volcano plot depicting differential metabolites. Each point on the plot corresponds to a different metabolite. The *x*-axis represents the logarithm of the fold change (FC), which is the quantitative difference in multiples of a metabolite before and after the intervention. The *y*-axis represents the variable of importance in the projection value, indicating statistical significance. Blue dots (FC < 1.5, *p*-value ≤ 0.05) denote downregulated differentially expressed metabolites, red dots (FC > 1.5, *p*-value ≤ 0.05) represent upregulated differentially expressed metabolites, and black dots indicate metabolites detected but not significantly different. The lower panel presents a lollipop chart to visualize the trends in these significant metabolites. In this chart, circle size and bar height both represent the degree of correlation, while color depth indicates the magnitude of the *p*-value. (**B**) Comparison of changes in lipoprotein subclasses before and after the probiotics intervention. Dot size represents the *p*-value of significance and dot color indicates the log2-fold change in expression within lipoprotein classes. Abbreviations: Gly, glycine; TG by PG, ratio of triglycerides to phosphoglycerides; Size_Liproprotein_Lipid_pct, the specific lipid-to-total lipids ratio in lipoprotein size subfractions (Size: XXL, extremely large; XL, extra-large; L, large; M, medium; S, small. Lipoprotein: VLDL, very low-density lipoprotein; HDL, high-density lipoprotein. Lipid: TG, triglyceride; C, cholesterol; CE, cholesteryl ester; PL, phospholipid). Statistically significant at *, *p* ≤ 0.05.

In the probiotics group, significant changes were observed in 30 metabolic parameters following the probiotic *L. bulgaricus* intervention. These changes included upregulation of several lipids (mainly C and CE) in HDL and VLDL, downregulation of several lipids (mainly TG and PL) in HDL and VLDL, and downregulation of total lipid and TG in VLDL of different sizes (Figure 3). In addition, metabolites of glycolysis, including lactate and acetate, were upregulated. Overall, there were significant improvements in the regulation of TG and lipid content in VLDL and HDL, which were not observed in the placebo group. These findings suggest the potential benefits of probiotic *L. bulgaricus* for overweight individuals with high blood TG levels.

### 3.5. Harms

No significant serious or moderate adverse events were found in the two groups.

## 4. Discussion

The World Obesity Federation’s prediction that more than half of the global population will be overweight or obese by 2035 [18] underscores the alarming and escalating public health issue of obesity worldwide. Research on the impact of probiotics on obesity contributes significantly to future public discussions on obesity control. Over the past few years, probiotics have gained recognition as a promising avenue for the development of therapeutic and preventative solutions for addressing metabolic syndrome. Several species of *Lactobacillus*, including *L. casei*, *L. fermentum*, *L. acidophilus*, *L. rhamnosus*, *L. paracasei*, and *L. bulgaricus*, have been documented to possess various probiotic functions, such as reducing blood lipid levels, safeguarding cardiovascular health, and mitigating the symptoms associated with obesity, in diet-induced obesity models [16,19,20,21,22]. The present study is the first to investigate the effects of *L. bulgaricus* on obesity control in human subjects. Our results show that following *L. bulgaricus* intervention, there was a significant decrease in TG levels, while no significant changes were observed in the body weight, body fat percentage, BMI, and major biochemical parameters (Table 2 and Table 3). High serum TG levels serve as a risk factor for cardiovascular diseases and play a role in the development of arteriosclerosis, thereby heightening the incidence of stroke, heart attack, and heart disease [23]. Therefore, while daily supplementation with *L. bulgaricus* may not lead to a reduction in body weight, it could potentially offer health benefits for obese individuals.

The metabolic profile analysis shows that daily supplementation with the probiotic, *L. bulgaricus*, had a positive effect by improving the lipoprotein lipid profile in overweight people. Lipoproteins serve as transport vehicles, allowing lipids to be carried throughout the body to various tissues and organs. The distribution, composition, and population of lipoproteins in the bloodstream, along with the dynamic processes they undergo (including synthesis, turnover, metabolism, and clearance), serve as crucial indicators for evaluating overall health. Dyslipidemia, characterized by a decreased C proportion in HDL and increased TG-rich lipoproteins, especially VLDL, is prevalent among obese individuals [24,25,26,27]. A decrease in TG levels in VLDL and an increase in the C proportion in HDL are generally associated with improved lipid metabolism, a reduced risk of cardiovascular diseases, and potential impacts on gut microbiota [28,29]. According to the metabolome data in the present study, *L. bulgaricus* supplementation reduced the TG proportion in lipoproteins while increasing the CE or C proportion, particularly within the lipoproteins VLDL and HDL. VLDL, specifically the TG-rich VLDL associated with hypertriglyceridemia, is acknowledged as a carrier of “bad” lipoproteins in the bloodstream. Elevated VLDL levels are linked to increased risks of various diseases, including obesity. In the physiological condition, VLDL functions as a cargo carrier, transporting cholesterol, TGs, and proteins to peripheral cells for essential bioactivities. Some studies suggested that VLDL not only serves as a lipid cargo carrier but also modulates lipid-related blood pressure regulation [28,30,31]. The observed alteration in lipid carrier content due to the *L. bulgaricus* probiotic supplement appears to be a positive sign for attenuating metabolic syndrome.

Microbiota metabolism may also be involved in the effect of the *L. bulgaricus* probiotic on metabolome changes in overweight people. The increase in glycolysis metabolites in obesity can be ascribed to two potential scenarios: (1) an excessive consumption of glucose and (2) the influence of probiotics. High glucose consumption can accelerate glycolysis and increase blood lactate and acetate levels, potentially resulting in a buildup of metabolic intermediates such as lipids and TG [32,33,34]. These metabolic intermediates may be capable of being converted to TG and being stored in VLDL particles, contributing to a high TG proportion in VLDL. In the present study, blood lactate and acetate levels increased in the probiotics group. However, the *L. bulgaricus* probiotic intervention led to a decrease In blood TG levels and a decrease in the TG proportion in VLDL and HDL. On the other hand, during the probiotic fermentation process, probiotics can produce lactate and short-chain fatty acids, including acetate, which can cause alterations in the gut microbiota composition and metabolite production in humans [34,35,36,37]. The increase in lactate and acetate may be associated with probiotic activity rather than high glucose consumption. Although previous studies have suggested that improvement of the composition of beneficial bacteria in the gut microbiota by probiotic supplementation may change lipid metabolism in humans [36,38,39,40], the mechanism of alteration of the lipid profile by *L. bulgaricus* needs further clarification in future studies.

Although the effect of *L. bulgaricus* supplementation on weight reduction has never been investigated clinically before, various species of *Lactobacillus* have been studied for weight management. Taking Puritan’s Pride (2.4 × 10^9^ CFU of *Lactobacillus*/day) for 6 months enhanced weight reduction in the participants with Roux-en-Y gastric bypass surgery [41]. Drinking fermented milk containing 5 × 10^10^ CFU *L. gasseri* STB2005 per day for 12 weeks decreased weight and fat mass in adults with obese tendencies [42]. Consuming light Yakult with *L. casei* Shirota (3 × 6.5 × 10^9^ CFU/day) for 12 weeks did not cause weight change in the patients with metabolic syndrome [43]. Taking *L. gasseri* BNR17 capsules (3 × 2 × 10^10^ CFU/day) for 12 weeks in overweight and obese adults without behavioral or dietary modifications (*n* = 31/group) did not induce weight changes but decreased the BMI and the waist and hip circumferences [11]. Consuming a capsule containing *L. acidophilus* CUL60, *L. acidophilus* CUL21, *L. plantarum* CUL66, and *Bifidobacterium animalis* subsp. *lactis* CUL34 (5 × 10^10^ CFU/day) for 9 months in overweight adults without behavioral or dietary modifications significantly decreased body weight and waist and hip circumferences [10]. Compared to these studies, 12 weeks is a common duration for evaluating weight reduction efficacy. However, we used a relatively low dosage of *L. bulgaricus* 1 × 10^8^ CFU/day in this clinical study, which is a regular dosage of commercial products for daily healthcare. On the other hand, for the effect of *Lactobacillus* on weight reduction in participants without diet and activity control, a long-term trial may provide benefits. It is possible that higher dosage treatment and long-term trials could present the weight reduction effect of *L. bulgaricus*. More studies are needed to clarify this.

In addition to metabolic regulation, previous research has identified numerous regulatory functions of *L. bulgaricus* that contribute to health. In mouse experiments, it was found to prevent colitis-associated cancer by inhibiting intestinal cytokines [44]. It has demonstrated immunoregulatory capabilities [45,46,47,48] suppressed lung inflammation in mice with asthma [49], and its exopolysaccharides were shown to inhibit influenza virus infection in lung cells [50]. *L. bulgaricus* fermented milk has exhibited robust antioxidant activity, alleviating alcohol-induced liver damage in mice [51]. Clinical trials have revealed that *L. bulgaricus* can enhance systemic immune function in the elderly [52], alleviate inflammation in atopic dermatitis [53], and improve various inflammation and oxidative stress biomarkers in women with gestational diabetes mellitus [54]. Moreover, daily consumption of yogurt fermented with *L. bulgaricus* has been shown to increase serum antibody titers, preventing influenza virus infection in elderly residents of nursing homes [55] and in healthy adults [56]. In summary, although the effects were often enhanced by selecting a specific strain of *L. bulgaricus*, it can still be anticipated that the intake of *L. bulgaricus* supplement provides various health benefits.

## 5. Limitation and Recommendation

In the present study, *L. bulgaricus* supplementation did not result in weight reduction over the course of 12 weeks. However, it is important to note that this trial served as a pilot study with a limited sample size. The small sample size may compromise the statistical significance of drug effects, particularly for treatments with milder efficacy. The probiotic group displayed a more severe lipid profile at baseline, potentially making weight reduction more challenging. Therefore, future trials with larger sample sizes will be necessary to thoroughly investigate the detailed effects of *L. bulgaricus* supplementation. The current study has limitations in drawing definitive conclusions. Furthermore, this trial had a relatively short duration of 12 weeks. Extending the study duration could offer valuable data on the longer-term effects of *L. bulgaricus* supplementation.

To improve the future studies, it is recommended to take more extensive investigations with large sample sizes. In the current study, the participants were instructed to maintain their regular diet and lifestyle, aiming to simulate real-world conditions. Based on the result of the present study, it is suggested that incorporating diet and activity control and implementing higher dosages of *L. bulgaricus* treatment may enhance its effectiveness in future research. For the study investigating *L. bulgaricus* efficacy under diet control, it would be a great addition to analyze the stool of the participants, including the microorganism composition and short-chain fatty acids levels. Exploring specific strains isolated from *L. bulgaricus* holds the potential for optimizing its efficacy. In large-scale trials, conducting subgroup analyses based on factors such as age, gender, and the severity of metabolic syndrome among participants could provide more detailed information about the precise efficacy of *L. bulgaricus* supplementation. These refinements and considerations may contribute to a more comprehensive understanding of the effects of *L. bulgaricus* in diverse populations and conditions.

## 6. Conclusions

This study discovered that daily supplementation with *L. bulgaricus* probiotics at a dose of 1 × 10^8^ CFU/day for 12 weeks did not result in weight reduction. Conversely, the supplementation led to a decrease in serum TG levels, potentially contributing to the reduction in TG content in lipoprotein composition. This effect may have a positive impact on preventing diseases associated with metabolic syndrome in overweight individuals. Further large-scale and long-term trials with higher dosage treatments are needed to clarify the effect of *L. bulgaricus* across various criteria for optimal application.

## Figures and Tables

**Table 1:** Baseline descriptive characteristics of participants of the subjects.

Group	Probiotics (*n* = 18)	Placebo (*n* = 13)	*p*-Value
	Mean ± SEM	Mean ± SEM	
Age, years	41.56 ± 2.573	41.38 ± 3.526	0.9042
Weight, kg	80.77 ± 3.561	78.69 ± 4.337	0.5888
Body fat, %	30.84 ± 1.240	32.08 ± 1.845	0.6742
BMI	28.89 ± 0.850	29.32 ± 1.499	0.8102

*p*-value Mann–Whitney test (compared probiotics and placebo group).

**Table 2:** Comparison of body weight, fat, and BMI between probiotics and placebo groups.

Variables		Probiotics (*n* = 18)	Placebo (*n* = 13)	
		Mean ± SEM	Mean ± SEM	*p*-value ^b^
Weight (kg)	Pre	80.77 ± 3.561	78.69 ± 4.337	0.2944
	Post	80.63 ± 3.559	77.97 ± 4.193	0.2945
	Change	−0.1389 ± 0.3988	0.7231 ± 0.8569	0.4443
	*p*-value ^a^	0.3660	0.2076	
Fat (%)	Pre	30.84 ± 1.24	32.08 ± 1.845	0.3371
	Post	31.27 ± 1.189	32.55 ± 1.690	0.3444
	Change	0.4278 ± 0.3195	0.4692 ± 0.5088	0.2479
	*p*-value ^a^	0.0991	0.1873	
BMI (kg/m^2^)	Pre	28.89 ± 0.8459	29.32 ± 1.499	0.4051
	Post	28.90 ± 0.8431	28.88 ± 1.291	0.3974
	Change	0.0056 ± 0.1620	−0.4385 ± 0.4160	0.3152
	*p*-value ^a^	0.4865	0.1563	

*p*-value ^a^: paired *t*-test (compared pre- and postgroup) and *p*-value ^b^: Mann–Whitney test (compared probiotics and placebo group).

**Table 3:** Comparison of serum biomedical parameters between probiotics and placebo groups.

Variables		Probiotics (*n* = 16–18)	Placebo (*n* = 12–13)	
		Mean ± SEM	Mean ± SEM	*p*-value ^b^
ALT (U/L)	Pre	32.00 ± 4.495	31.00 ± 6.454	0.7219
	Post	28.12 ± 4.853	37.69 ± 7.946	0.5161
	Change	−3.882 ± 3.461	6.692 ± 6.747	0.0738
	*p*-value ^a^	0.1392	0.1704	
AST (U/L)	Pre	24.31 ± 1.886	22.38 ± 3.054	0.2812
	Post	22.44 ± 2.238	25.85 ± 4.366	0.8429
	Change	−1.875 ± 1.530	3.462 ± 2.043	0.0147 *
	*p*-value ^a^	0.1196	0.0580	
BUN (mg/dL)	Pre	13.82 ± 0.6765	13.17 ± 0.8242	0.5034
	Post	12.88 ± 0.8985	12.67 ± 0.7914	0.9626
	Change	−0.6250 ± 0.7465	−0.500 ± 0.812	0.4533
	*p*-value ^a^	0.2078	0.2753	
CRE (mg/dL)	Pre	0.8241 ± 0.03584	0.7962 ± 0.0443	0.6006
	Post	0.8775 ± 0.04979	0.7915 ± 0.04273	0.245
	Change	0.0475 ± 0.02713	−0.0046 ± 0.0209	0.1131
	*p*-value ^a^	0.0502	0.4143	
CHOL (mg/dL)	Pre	200.7 ± 10.33	186.9 ± 11.49	0.5578
	Post	204.6 ± 12.42	186.3 ± 10.20	0.4766
	Change	3.882 ± 7.086	−0.6154 ± 3.852	0.3610
	*p*-value ^a^	0.2957	0.4379	
Glu-AC (mg/dL)	Pre	109.3 ± 7.834	91.77 ± 4.590	0.0847
	Post	110.7 ± 9.383	91.69 ± 6.466	0.2138
	Change	1.389 ± 2.332	−0.077 ± 2.962	0.2171
	*p*-value ^a^	0.2797	0.4899	
HbA1c (%)	Pre	6.124 ± 0.2559	5.800 ± 0.2295	0.4476
	Post	6.407 ± 0.3149	5.931 ± 0.3175	0.1878
	Change	0.2067 ± 0.0589	0.131 ± 0.098	0.0703
	*p*-value ^a^	0.0017 *	0.1027	
Insulin (μIU/mL)	Pre	14.400 ± 5.303	9.443 ± 1.610	0.6168
	Post	9.670 ± 1.591	9.208 ± 1.439	0.9001
	Change	−5.040 ± 4.652	−0.235 ± 0.943	0.0513
	*p*-value ^a^	0.1473	0.4035	
HDL-C (mg/dL)	Pre	48.55 ± 3.820	46.82 ± 1.613	0.3151
	Post	47.38 ± 3.125	46.43 ± 1.798	0.3925
	Change	−0.0125 ± 1.362	−0.392 ± 1.590	0.4218
	*p*-value ^a^	0.4964	0.4046	
LDL-C (mg/dL)	Pre	113.0 ± 7.399	122.8 ± 12.17	0.4925
	Post	119.0 ± 5.992	110.8 ± 8.862	0.7064
	Change	6.012 ± 4.366	−12.04 ± 6.323	0.0244 *
	*p*-value ^a^	0.0937	0.0417 *	
TG (mg/dL)	Pre	220.9 ± 42.18	139.6 ± 14.47	0.1608
	Post	199.9 ± 40.46	177.4 ± 36.55	0.6008
	Change	−21.00 ± 11.61	37.77 ± 34.12	0.0412 *
	*p*-value ^a^	0.0447 *	0.1450	
Alb (g/dL)	Pre	4.541 ± 0.06700	4.415 ± 0.07057	0.1031
	Post	4.656 ± 0.3614	4.485 ± 0.07412	0.1793
	Change	0.125 ± 0.06423	0.0692 ± 0.0624	0.3287
	*p*-value ^a^	0.0353 *	0.1445	
CRP (mg/dL)	Pre	0.2441 ± 0.04415	0.2577 ± 0.07466	0.6753
	Post	0.2231 ± 0.04520	0.1800 ± 0.04403	0.6594
	Change	−0.0200 ± 0.03423	−0.0777 ± 0.0440	0.0933
	*p*-value ^a^	0.2839	0.0513	

*p*-value ^a^: paired *t*-test (compared pre- and postgroup) and *p*-value ^b^: Mann–Whitney test (compared probiotics and placebo group). Statistically significant at *, *p* ≤ 0.05.

## Data Availability

The raw data supporting the conclusions of this article will be made available by the authors on request. Data is not publicly available due to privacy.

## Supplementary Materials

The following supporting information can be downloaded at: https://www.mdpi.com/article/10.3390/metabo14020129/s1, Figure S1: Differential metabolites identified in baseline lipid profiling.
