# Supplementary material for: The Efficacy of Lactobacillus delbrueckii ssp. bulgaricus Supplementation in Managing Body Weight and Blood Lipids of People with Overweight: A Randomized Pilot Trial
Source: Metabolites. 2024 Feb 16;14(2):129. doi: 10.3390/metabo14020129 (PMC10890272; doi:10.3390/metabo14020129)
Supplement: Supplementary file 1 [file metabolites-14-00129-s001.zip › metabolites-2756114-supplementary.pdf]

metabolites

Article

The Efficacy of *Lactobacillus delbrueckii* ssp. *bulgaricus* Supplementation in Managing Body Weight and Blood Lipids of People with Overweight: A Randomized Pilot Trial

Pei-Yi Chu [1], Ying-Chun Yu [2,3], Yi-Cheng Pan [1,4], Yun-Hao Dai [1,5], Juan-Cheng Yang [1,6], Kuo-Chin Huang [7,8,*] and Yang-Chang Wu [1,9,10,*]

[1] Chinese Medicine Research and Development Center, China Medical University Hospital, Taichung, Taiwan; ositachucmu@gmail.com (P.-Y.C.); alce2943@gmail.com (Y.-C.P.); s8355099@gmail.com (Y.-H.D.); qq9113054@gmail.com (J.-C.Y.)
[2] Department of Medical Research, Department of Obstetrics and Gynecology, Department of Gastroenterology, and Chinese Medicine Research and Development Center, China Medical University Hospital, Taichung, Taiwan; yingchun.ycyu@gmail.com
[3] Graduate Institute of Biomedical Sciences, Center for Tumor Biology, School of Medicine, China Medical University, Taichung, Taiwan
[4] Ph.D. Program for Cancer Biology and Drug Discovery, China Medical University and Academia Sinica, Taichung, Taiwan
[5] School of Pharmacy, China Medical University, Taichung, Taiwan
[6] School of Chinese Medicine, College of Chinese Medicine, China Medical University, Taichung, Taiwan
[7] Department of Chinese Medicine, China Medical University Hospital, Taichung, Taiwan
[8] School of Chinese Medicine, China Medical University, Taichung, Taiwan
[9] Graduate Institute of Integrated Medicine, College of Chinese Medicine, China Medical University, Taichung, Taiwan
[10] Department of Medical Laboratory Science and Biotechnology, College of Medical and Health Science, Asia University, Taichung, Taiwan
* Correspondence: 004322@tool.caaumed.org.tw (K.-C.H.); yachwu@mail.cmu.edu.tw (Y.-C.W.)

Supplementary data

Subjects' Lipid Profile Characteristics

Upon analyzing the baseline metabolome data, significant disparities in specific lipid parameters were observed between the groups. A DE analysis conducted on lipoprotein subclasses revealed that the probiotics group exhibited higher baseline levels of the proportion of TG in extremely large very low-density lipoproteins (XXL_VLDL), the proportion of phospholipids (PL) in large VLDL (L_VLDL), concentration of very small VLDL (XS_VLDL) particles, and tyrosine (Try) and lower levels of the degree of unsaturation fatty acids, the ratio of polyunsaturated fatty acids (PUFA) to total fatty acids, and the ratio of docosahexaenoic acid (DHA) to total fatty acids compared to the placebo group (Figure S1).

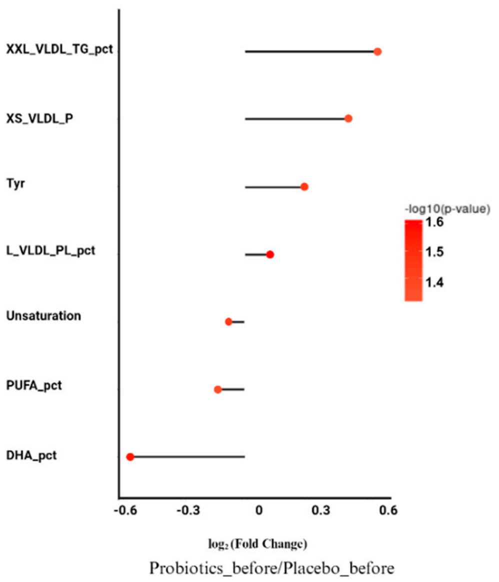

Figure S1. Differential metabolites identified in baseline lipid profiling. Lollipop chart shows the log (fold change) of comparing lipid metabolites in participants' baseline between probiotics and placebo groups (probiotics before vs. placebo before). The size of the circle and the length of the bar convey the degree of correlation, while the color depth indicates the magnitude of the p-value. (XXL_VLDL_TG pct, TG to total lipids ratio in chylomicrons and extremely large VLDL; XS_VLDL_P, concentration of extra-small VLDL particles; Tyr, tyrosine; L_VLDL_PL_pct, phospholipids to total lipids ratio in large VLDL; Unsaturation, degree of fatty acid unsaturation; PUFA_pct, ratio of polyunsaturated fatty acids to total fatty acids; DHA_pct, ratio of docosahexaenoic acid to total fatty acids).
